# Research on the coupling mechanism and influencing factors of digital economy and green technology innovation in Chinese urban agglomerations

**DOI:** 10.1038/s41598-024-55854-4

**Published:** 2024-03-02

**Authors:** Xuesi Zhong, Ziyi Duan, Chang Liu, Wei Chen

**Affiliations:** 1 School of Economics and Management, Guangxi Normal University, Guilin, 541000 Guangxi China; 2 Pearl River-Xijiang River Economic Belt Development Institute, Guangxi Normal University, Guangxi, 541004 Guilin China; 3 Business School, Guilin Tourism University, Guilin, 541006 Guangxi China

**Keywords:** Digital economy, Green technology innovation, Coupling coordination, Urban agglomeration, Socioeconomic scenarios, Sustainability

## Abstract

This paper examines the coupling coordination degree between digital economy and green technology innovation in 19 urban agglomerations across China from 2011 to 2020. Through the analysis of the coupling coordination degree model, spatial autocorrelation, multi-distance spatial clustering analysis, kernel density analysis and grey correlation model, this study uncovers the mechanism of coupling between digital economy and green technology in Chinese urban agglomerations. Data analysis revealed a significant increase in the coupling coordination between the digital economy and green technology innovation within urban agglomerations. However, there are noticeable spatial imbalances in this trend. Additionally, the multi-distance spatial distance analysis highlights a shift from a random distribution to a clustered distribution of spatial characteristics. The polarization features vary among each urban agglomeration and exhibit a significant positive spatial correlation. Factors such as economic sustainability, creative talent, policy support, digital impetus, and technological support will affect the coupling mechanism of green technology innovation and the digital economy in China's urban agglomerations. Policy recommendations are proposed to foster the development of the digital economy, promote coordinated growth within and beyond urban clusters, and ultimately build a digital ecological civilization that is both green and intelligent.

## Introduction

In recent years, with the rapid development of big data, cloud computing, 5G and other digital technologies, the digital economy has become an important engine of economic development for countries around the world. At the same time, the call for green transformation of the global economy has intensified due to the severe environmental pollution problem. The digital economy has a green value, which can improve green technological innovation and resource allocation efficiency^1^; however, digital infrastructure as the carrier of digital economic development is not always green, and is often labelled as "energy giants"^2^. Therefore, it is urgent to vigorously develop green technological innovation; the digital economy and green development constrain each other^3^. Therefore, how to promote the coordinated development of digital economy and green technological innovation is an urgent issue that deserves attention. The 2022 Digital China Development Report released by the National Internet Information Office shows that the scale of the digital economy has reached 50.2 trillion yuan, accounting for 41.5% of GDP; China's gross national product has increased from 0.4 trillion yuan in 1978 to 121 trillion yuan in 2022, and its total economic output has jumped to the second place in the world. However, with the rapid growth of China's economy, the crude economic development method has gradually revealed problems such as low efficiency and environmental pollution that need to be solved. The Chinese government has proposed a dual-carbon target at the 2020 UN General Assembly, signalling China's desire to follow a green and sustainable development path and to vigorously promote green technological innovation. This shows that it is highly representative to use China as a role model to explore the driving path of coordinated development of the digital economy and green technological innovation, and to provide practical experience for other developing countries.

Based on existing research, it has been found that domestic and foreign scholars have roughly divided their investigations into the relationship between digital economy and green technology innovation into three directions. The potential positive or negative effects of the digital economy on green technology innovation are explored using fixed panel, threshold effect, spatial Dubin and other models, from a one-way effect perspective. Abbreviations of technical terms will be explained in their first usage. For instance, there exists a nonlinear correlation between digital economy and green technology innovation^4,5^. And the digital economy can optimize the energy structure and promote the quality and quantity of green technological innovation in enterprises^6^, Zhang et al.^7,8^ with significant spatial spillover effects^9,10^. Moreover, the regional heterogeneity displayed two decreasing trends of "increasing first and then decreasing" and "decreasing" (excluding Xinjiang)^11^. Furthermore, environmental regulation tools can affect the regulatory impact of the digital economy on green technology innovation. The digital economy can also indirectly impact the CGTI through intermediary variables such as environmental regulation, marginal effect, and spatial spillover effect^12^. The digital economy encourages innovation in green technology by facilitating the structural transformation of the secondary industry. However, transforming the primary and tertiary industries may lead to reduced efficiency in green technology innovation^13^. Additionally, some scholars consider the digital economy or green technology innovation as an intermediary and introduce a third variable to study their influence and mechanisms of action. For instance, certain scholars utilise green technology innovation as a means to analyse the effect of the digital economy on the enhancement of industrial framework, green total factor productivity, and other related components. The digital economy is assumed to improve green total factor productivity by augmenting the level of green technology innovation^14,15^, as well as the upgrading of industrial structure^16^. It contributes to the advancement of sustainable and high-quality economic development, offering indicators such as the application of green practices, differentiated development, and collaborative digital economy development for achieving environmentally sound and superior industrial growth^17,18^. Additionally, certain scholars view the digital economy as a transmission mechanism and apply quantitative analysis to substantiate the potential of digital government in advancing the level of green technology innovation by propelling the growth of the digital economy^19^. Furthermore, examining the sub-division industry and micro performance of the digital economy, this paper examines its impact on the innovation of green technology. Some scholars have categorized industries in the context of the digital economy, encompassing areas such as green finance^20^, digital finance^21–23^, Internet development^24^, smart cities^25^, and e-commerce^26^. These scholars have investigated the mechanism of segmenting industries on the innovation of green technology^27^, threshold effect^28^, spatial effect^29^, and other related topics. Some researchers utilise the Dubin model, coupling coordination degree model, and exploratory spatial analysis method to examine the impact of digital economic industry segmentation on green technology innovation. They also measure the degree of coupling coordination development and spatial effect between the two^30^.

In summary, the academic community has carried out many useful explorations in terms of direct role, mediating role and sub-dimensions. However, it is found that (1) most of the existing studies have explored the one-way impact of the digital economy on green technology innovation, but neglected to assess the two-way interaction between the two, and the studies on the digital economy and green technology innovation have emphasised the use of mathematical models to carry out quantitative analyses, but do not have a cohesive theoretical sorting framework. (2) The scope of existing research on the coupling effect of the two is relatively narrow, and the exploration of the deeper reasons and driving forces behind the differences in coupling coordination needs to be extended. The research methods mainly focus on the measurement of the coupling development level, heterogeneity and spatial effect assessment, and most of them rely on the Moran index and exploratory spatial analysis methods, lacking more three-dimensional and intuitive research methods. (3) Most of the existing studies are based on the samples of prefecture-level cities or a single urban agglomeration, and have not been extended to the perspective of a national urban agglomeration to construct the evaluation index system of digital economy and GTI.

Its potential marginal contributions are as follows: Firstly, in terms of theoretical sorting, existing studies have not formed a unified theoretical framework for the relationship between digital economy and green technology innovation in city clusters. This paper tries to clarify the mechanism of coupled and coordinated development of digital economy and green technology innovation in city clusters, construct the evaluation index system of digital economy and green technology innovation under the perspective of city clusters, and incorporate the theoretical logic of existing studies into this theoretical framework. Secondly, in terms of research content, existing studies mainly focus on the single dimension of digital economy and green technology innovation, this paper uses multi-dimensional methods such as multi-distance spatial clustering analysis and kernel density analysis to explore the spatial and temporal evolution of the coupling degree of coordination between the digital economy and green technology innovation; and uses the grey correlation model to analyse the main factors affecting the degree of coordination of the coupling degree of urban agglomerations.

Finally, in terms of practical significance, existing studies have mainly focused on the exploration of single city clusters or prefecture-level cities across the country. This paper takes the 19 major city clusters in the country as samples for empirical research, which provides new empirical evidence for further promoting the coupled and coordinated development of digital economy and green technology innovation, and has certain practical value for promoting the formation of China to build the coordinated development of large, medium-sized and small cities relying on city clusters and metropolitan areas, and provides a new basis for promoting the high-quality development of China's digital economy in the new era.

## Theoretical analysis

**Figure 1:**
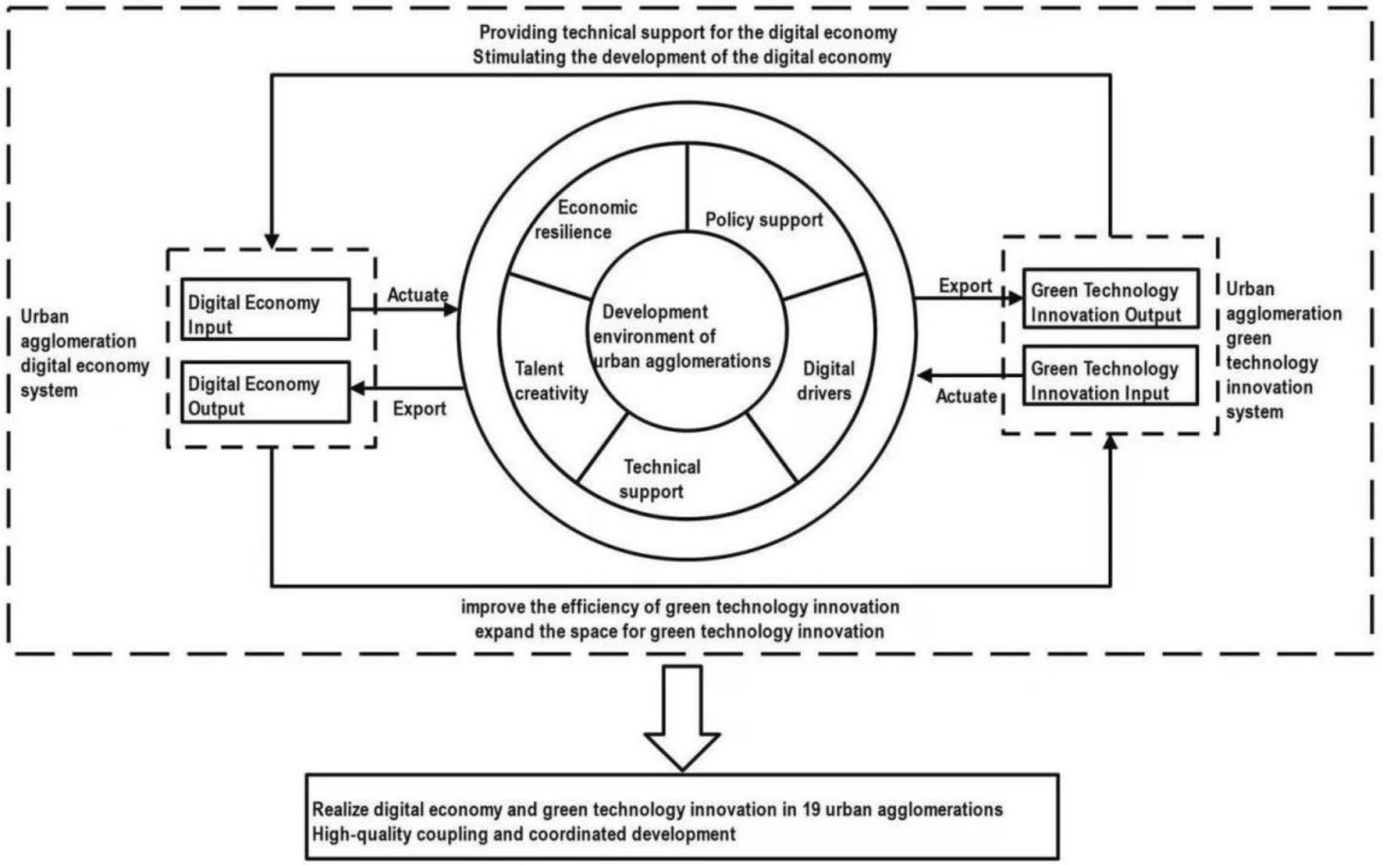
Coupling and coordinated development of digital economy and green technology innovation in urban agglomeration.

The digital economy regards data resources as its key element, with the modern information network functioning as its main body. It harnesses communication technology and all-factor digital transformation to establish a fairer and more efficient market environment^31^. A mutually reinforcing coupling relationship exists between the digital economy and green technology innovation. On one hand, the digital economy is a critical stimulus for the innovation of green technology; and on the other hand, green technology innovation offers significant support for the rapid advancement of the digital economy (refer to Fig. 1).

A mutually reinforcing coupling relationship exists between the digital economy and green technology innovation. The digital economy has increased the value-added and improved the efficacy of innovation in green technology^32^. Initially, the digital economy alleviates financial limitations for projects affiliated with green technology innovation. The rapid advance of the digital economy has fostered new forms of commerce like digital finance and inclusive finance, it has also engendered various forms of internet finance, such as internet banking and P2P online credit, which have effectively mitigated the financial limitations faced by small and medium-sized enterprises due to traditional market financing. These developments provide more options for financing green technology innovation projects^33^. Secondly, the digital economy promotes the inclination for innovation among green enterprises. It unites various fragmented market segments and eliminates constraints such as transaction time and geographical distance. This leads to a more frequent flow of production factors and consumer demands from all over the world on digital platforms and ultimately diminishes the original geographic advantages of enterprises^34^. Third, the digital economy lowers the controllable expenses of Global Trade Integration (GTI). It allows companies to analyse market trends and allocate production factors according to demand. Innovation stakeholders can utilise digital technology to identify and exchange resources, thereby reducing resource acquisition costs^35^. Additionally, the digital economy strengthens the reliability of GTI outcomes. Contemporary internet, big data and other communication technologies employ mathematical models and algorithms to identify innovation decisions aligned with enterprise conditions and development goals, thereby expediting the transformation of achievements.

Green technology innovation lends technical support and demand pull to the digital economy's development. GTI offers technical support for the digital economy's development from a technological standpoint. The development of digital economy infrastructure, encompassing telecom and network infrastructure, requires support from innovative technology, personnel, and capital. Improvement in the quality of digital infrastructure construction and promotion of digital economy development can be achieved through technological and model innovations^36^. Second, examining the economic and environmental impact, GTI generates a favourable external environment for the growth of the digital economy. The outcome of developing green technology is the enhancement of both economic advantages and environmental quality, facilitating the transition towards the supply-side, while digital transformation serves as an essential measure for businesses to attain self-transformation and achieve high-quality development^37,38^.

There is a mutually beneficial relationship between digital economy and green technology innovation, leading to an overall increase in coordinated development. Firstly, economic development acts as a fundamental prerequisite for green technology innovation and the integration of digital technology into various scenarios^39^. Secondly, the pursuit of stable, high-quality, and sustainable economic growth is a significant objective of macro-control measures. Strong policy support and adjustment will provide institutional guarantees for their coordinated development. Moreover, the levels of digitalisation and technology are significant driving forces in promoting coordinated development between the two. These forces not only enhance the development of digital economy and green technology innovation, but also forge new forms of coupling and coordinated development, thereby paving the way for practical approaches to fostering coordinated development of the two. Fourthly, skilled individuals are the fundamental asset and those possessing high-level expertise hold an upper hand in strategic planning, management of innovation, and research and development of technology^40^.

## Research design

### Sample selection

According to the development planning documents of 19 urban agglomerations that have been clearly planned and constructed by The State Council and the National Development and Reform Commission, the research scope has been established^41^ (See Table 1).

**Table 1:** Division of 19 urban agglomerations.

According to the development plan of different division	Urban agglomeration
Five national urban agglomerations	Yangtze River Delta, Pearl River Delta, Beijing-Tianjin-Hebei, Middle reaches of Yangtze River, Chengdu-Chongqing
Eight regional urban agglomerations	South Central Liaoning, Shandong Peninsula, Guangdong, Fujian and Zhejiang coastal areas, Harbin-Changjiang, Central Plains, Guanzhong Plain, Beibu Gulf, Tianshan North Slope
Six regional urban agglomerations	Jinzhong, Hubao EYu, Central Yunnan, Central Guizhou, Lanxi, Ningxia along the Yellow River

This study utilises spatial panel data from 2011 to 2020 to assess the coupling and coordination of the digital economy and green technology innovation system, or TG system, within China's urban agglomerations. After screening and eliminating data, a total of 200 prefecture-level cities were selected as research samples for urban clusters. This decision was made due to overlapping data samples and severe data insufficiency in certain cities. The data pertaining to each region is derived from the China Statistical Yearbook, China City Statistical Yearbook, and the Statistical Bulletin, spanning from 2011 to 2021. In instances where data was missing, the official website of the National Bureau of Statistics of China and the provincial and regional statistical offices was consulted, and linear imputation was utilized where necessary.

### Research methods

#### Entropy weight TOPSIS method

The entropy weight method, originally proposed by Claude Elwood Shannon, is extended through the traditional approach. TOPSIS assesses all evaluated objects by computing the relative distance from the optimal and worst solutions. The entropy weight-TOPSIS method provides flexible and consistent evaluation results^42^. This paper employs the entropy weight TOPSIS approach within MATLAB software to determine the weight for each index of the digital economy and innovation in green technology.

#### Coupling degree and coupling coordination degree model

The coupling coordination degree model is used to analyze the level of coordinated development of things. The degree of coupling reflects the degree of interdependence and mutual constraints between systems. The degree of coordination refers to the size of the degree of benign coupling in the mutual coupling relationship, which can reflect the good or bad coordination status. The coupling degree of digital economy and green technology innovation is to measure the degree of interaction, interdependence and mutual constraints between the two systems of "digital economy" and "green technology innovation", and to verify the level of this interactive effect. By creating a model of the coupling and coordination degrees of digital economy and green technology innovation (TG system) in urban agglomerations, this paper evaluates the coordination status of these systems in China. The model construction is illustrated in Formulae (1), (2) and (3).

1 $$ C = \sqrt[2]{{S_{1}^{\prime} S_{2}^{\prime} /\left( {S_{1}^{\prime} + S_{2}^{\prime} } \right)}} $$

2 $$ T = \alpha S_{1}^{\prime} + \beta S_{2}^{\prime} $$

3 $$ D = \sqrt {CT} $$

In the equation, $S_{1}^{\prime}$ and ${\text{S}}_{2}^{\prime}$ represent the overall index of digital economy and green technological innovation of urban clusters, respectively. C (0 ≤ C ≤ 1) denotes the degree of coupling in the TG system, and the nearer C approaches 1, the greater the degree of coupling. T is the coordination index, while α and β respectively signify the weight of the two subsystems in the evaluation index of the TG system. D (0 ≤ D ≤ 1) represents the complete evaluation index of the TG system's coupling and coordination development level. Based on Coupling Coordination research^43,44^, the various types of TG system coupling degrees and coordination degrees are classified into seven different stages, as provided in Table 2.

**Table 2:** Judgment of coupling degree, coupling coordination degree and classification of discrimination relation.

Coupling degree judgment	Coupling phase	Coupling coordination degree judgment	Coupling coordination level	System index comparison	Relational discrimination feature
C = 0	Coupled independence	0 ≤ D < 0.2	Severe dysregulation	0 < $S_{1}^{\prime } /{\text{S}}_{2}^{\prime }$ ≤ 0.3	Green technology innovation lags behind
0 < C < 0.1	Coupling start	0.2 ≤ D < 0.3	Moderate dysregulation	0.3 < $S_{1}^{\prime } /{\text{S}}_{2}^{\prime }$≤ 0.5	Green technology innovation generally lags behind
0.1 ≤ C < 0.3	Coupling antagonism	0.3 ≤ D < 0.4	Mild disorder	0.5 < $S_{1}^{\prime } /{\text{S}}_{2}^{\prime }$ ≤ 0.8	Green technology innovation lags slightly
0.3 ≤ C < 0.5	Coupling running-in	0.4 ≤ D < 0.5	Borderline disorder	0.8 < $S_{1}^{\prime } /{\text{S}}_{2}^{\prime }$ ≤ 1.2	Basic synchronization
0.5 ≤ C < 0.8	Coupling growth	0.5 ≤ D < 0.6	Primary coordination	1.2<$S_{1}^{\prime } /{\text{S}}_{2}^{\prime }$ ≤ 2.0	The digital economy is lagging slightly
0.8 ≤ C < 1.0	Coupling maturation	0.6 ≤ D < 0.7	Intermediate coordination	2.0 < $S_{1}^{\prime } /{\text{S}}_{2}^{\prime }$ ≤ 3.0	The digital economy generally lags
C = 1.0	symbiosis	0.7 ≤ D ≤ 1.0	Advanced coordination	$S_{1}^{\prime } /{\text{S}}_{2}^{\prime }$ > 3.0	The digital economy is lagging badly

#### Spatial autocorrelation

The purpose of conducting a spatial autocorrelation analysis is to determine if there is a correlation between a specific attribute of a ground object and the attribute of the object in the surrounding space, as well as the strength of this correlation^45^. This paper utilises ArcGIS 10.2 software to extract and convert data on the coupling coordination degree between urban agglomeration digital economies and green technology innovation. Furthermore, the spatial autocorrelation analysis tool is employed to investigate the spatial autocorrelation of this data across several years.

#### Multi-distance spatial clustering analysis

Ripley's K function analysis considers the coupling coordination degree of the digital economy and green technology innovation in every urban agglomeration as a spatial point and assesses the clustering degree of point data sets at varying distances to indicate the level of spatial aggregation or dispersion of the coupling coordination degree of the digital economy and green technology innovation in urban agglomeration and its changes with varying neighbourhood sizes. Using Arcgis10.2 software's multi-distance spatial clustering analysis tool, the coupling coordination degree of digital economy and green technology innovation was analyzed in the 19th urban agglomeration. This was done through the Ripley's K function analysis and significance test, with point location maps being evaluated for each year. The calculation formula is as follows^46^:

5 $$ L\left( d \right) = \sqrt {\frac{K\left( d \right)}{\pi } - d} ;K\left( d \right) = \frac{A}{{n^{2} }}\mathop \sum \limits_{i}^{n} \mathop \sum \limits_{j}^{n} w_{ij} \left( d \right) $$

where n represents the number of 19 major cities; d is the distance scale; wij(d) represents the distance between two urban agglomerations, and A represents the area of the study area. If the value of L (d) is above the confidence interval, the coupling coordination degree of the TG system in the urban agglomeration displays significant spatial agglomeration characteristics. When L (d) falls within the confidence interval, the coupling coordination of the TG system within urban agglomerations displays a significant, randomly distributed spatial pattern.

#### Nuclear density analysis

Kernel density estimation is a non-parametric test method used in probability theory to estimate unknown density functions. Kernel density estimation is the estimation of the density parameter of a random variable by means of a kernel function, and the basis of nonparametric estimation constitutes the general logic of nonparametric estimation of fitted regression equations. The kernel function is used as a weighting function to correspond to the sample points in order to constitute a fitted estimate of the regression relationship^47^. In this paper, we use the kernel density estimation method to analyse the distribution dynamics of the coupling coordination degree between digital economy and green technology innovation in urban agglomerations during the observation period. To calculate the density estimation for data $w_{1}$, $w_{2}$, …, $w_{{\text{u}}}$, the kernel density estimation formula is applied:

6 $$ f_{h} \left( w \right) = \frac{1}{uh}\mathop \sum \limits_{i = 1}^{n} K\left( {\frac{{w - w_{i} }}{h}} \right) $$

where the kernel function K is a weighting function, and the Gaussian function is used in this paper. u represents the number of samples; h is bandwidth; $$w - w_{i}$$ Indicates the distance between $$w $$ and $$ w_{i}$$.

#### Geographic detector model

This essay investigates the impact of various factors on the spatial differentiation of the coupling coordination degree of the TG system in Chinese urban agglomerations using the factor detector and the interaction detector in the geographical detector.

The factor detector assesses the extent of the impact of the independent variable (X) on the dependent variable (Y). The driving effect is usually presented in statistical terms and calculated as follows:

7 $$ {\text{q}} = 1 - \frac{{\mathop \sum \nolimits_{h = 1}^{L} Nh\sigma h^{2} }}{{N_{\sigma }^{2} }} $$

where the objective is to assess the detection and explanatory power of influence factors on the coordination degree of TG systems. The study area is stratified by factors, and the total number of samples is represented by = 1,2,… The variance value of the overall sample in the study area is σ^2^. The value range is [0,1], where a larger value indicates a stronger driving effect of the independent variable (X) on the dependent variable (Y), and vice versa^48^.

The interaction detector assesses whether the interaction of any two non-independent factors, for instance, X_1_ and X_2_, amplifies or weakens their respective explanatory power for the dependent variable Y. You can find specific interactions and their criteria in Table 3.

**Table 3:** Geo-detector interactions and their criteria.

Criterion of criteria	Interaction effect
$q\left( {A \cap B} \right) < {\text{min}}\left[ {q\left( {\text{\rm A}} \right),q\left( {\text{B}} \right)} \right]$	Attenuation of nonlinearity
${\text{min}}\left[ {q\left( A \right),q\left( B \right)} \right] < q\left( {A \cap B} \right) < {\text{max}}\left[ {q\left( A \right),q\left( B \right)} \right]$	The single-factor nonlinearity decreases
$q\left( {A \cap B} \right) > {\text{max}}\left[ {q\left( {\text{\rm A}} \right),q\left( {\text{B}} \right)} \right]$	Two-factor enhancement
$q\left( {A \cap B} \right) = q\left( {\text{\rm A}} \right) + q\left( {\text{B}} \right)$	independent
$\left( {A \cap B} \right) > q\left( {\text{\rm A}} \right) + q\left( {\text{B}} \right)$	Nonlinear enhancement

### Construction of evaluation index system

By referring to relevant studies^49–51^ and combining with the actual development of the 19th Urban agglomeration, this paper comprehensively considers the principles of scientificity, systematicness, robustness and data availability of the indexes to construct the TG system evaluation index system of urban agglomeration. (See Table 4).

**Table 4:** Comprehensive evaluation system of TG system in urban agglomeration.

	Primary index	Secondary index	Three-level index	Nature	Unit
Digital economy system of urban agglomerations	Digital economy Put into	infrastructure	Mobile phone user	+	Ten thousand households
			Internet broadband access users	+	A hundred people
		Human resources	Number of employees in the information transmission, computer services and software industries	+	People
	The Digital Economy Output of goods	Basic industries	Revenue from telecommunications services	+	Ten thousand yuan
		Application industry	Breadth of digital coverage of inclusive finance	+	/
			Financial Inclusion Digitization Index	+	/
			Depth of digital use of inclusive finance	+	/
Green technology innovation system for urban agglomerations	Quality of green technology innovation	Number of green invention patent applications	Green invention patent	+	People
	Quantity of green technology innovation	Number of green patent applications	Green utility model patent	+	People
			Green design patent	+	People
			Green invention patent	+	People

## Empirical results and analysis

### Comprehensive evaluation of digital economy system

The paper employs the entropy weight method to calculate the comprehensive score of the digital economy for each city. Subsequently, the paper obtains the comprehensive score ranking of digital economies of urban agglomerations using weighted average results. The resulting grades are distributed in proportion (see Table 5).

**Table 5:** Comprehensive score and classification of digital economy system in urban agglomerations of China.

Urban agglomeration	Year				Total score	Classification of grades
	2011	2014	2017	2020		
Pearl River Delta	0.185	0.290	0.283	0.268	0.256	High
The Yangtze River Delta	0.149	0.211	0.217	0.212	0.197	High
The Beijing-Tianjin-Hebei	0.122	0.210	0.227	0.216	0.194	High
Guangdong, Fujian and Zhejiang coastal	0.120	0.181	0.191	0.197	0.172	Higher up
Shandong Peninsula	0.110	0.176	0.180	0.176	0.161	Higher up
Chengyu	0.094	0.161	0.183	0.174	0.153	Higher up
Hubao Eyu	0.099	0.155	0.159	0.177	0.148	Higher up
South Central Liaoning	0.099	0.157	0.160	0.145	0.140	Higher up
Jinzhong	0.089	0.148	0.158	0.152	0.137	Medium
Guanzhong Plain	0.081	0.147	0.160	0.155	0.136	Medium
Ningxia along the yellow	0.073	0.142	0.149	0.145	0.127	Lower
Central plains	0.072	0.127	0.142	0.156	0.124	Lower
Middle reaches of Yangtze River	0.075	0.131	0.141	0.137	0.121	Lower
Harbin-changjiang	0.075	0.124	0.138	0.125	0.116	Lower
Beibu Gulf	0.065	0.099	0.107	0.099	0.093	Low
in central Yunnan	0.057	0.094	0.098	0.093	0.086	Low
Lanxi	0.046	0.087	0.094	0.104	0.083	Low
Central Guizhou	0.043	0.089	0.081	0.094	0.077	Low
The north slope of Tianshan Mountain	0.045	0.046	0.051	0.052	0.048	Low

Overall, the digital economy in all urban agglomerations saw an initial upward trend from 2011 to 2020, followed by a downward trend in some urban areas in 2020. These findings are consistent with the fact that while the foundation of China's digital economy is relatively solid in 2011–2020, but it still faces structural imbalances, limited core technologies, inadequate implementation and a lack of institutions^52^. Since the outbreak of COVID-19 in 2020, the swift growth of urban demand for the digital economy has hastened the pace of digital economy development. Nevertheless, the uneven, insufficient, and non-standardised advancement of the digital economy remains a notable issue.

From 2011 to 2020, the local development trends of urban agglomerations share some similarities, however, the degree of development varies greatly. Notably, the Pearl River Delta urban agglomeration, which has the highest comprehensive score, has a difference of 0.208 as compared to the North Slope of Tianshan Mountain urban agglomeration with the lowest score. The Pearl River Delta, Yangtze River Delta, and Beijing-Tianjin-Hebei clusters attained top rankings in the final score of digital economy development among the 19 major city clusters. This empirical outcome is consistent with the earlier study by Luo and Zhou^53^. With a strong economic foundation and comprehensive policies and measures, Guangdong, Fujian, and Zhejiang coastal urban agglomerations have emerged as pilot zones for China's digital economy. The high digital economy score of these regions can be attributed to the comparative advantages in geographical location, industrial structure, and other factors enjoyed by the Northern Slope of Tianshan Mountain urban agglomeration, which provides a favorable development environment for the digital economy. The Chengdu-Chongqing urban agglomeration is connected to both the east and the west, featuring vast hinterlands and ample market space. It has emerged as a significant location for the sprawling development of the digital economy from the coast to the inland regions, exhibiting a high level of digital economic growth. The level of digital economic development in the urban agglomerations in eastern and central China is relatively advanced, whereas that in the western urban agglomerations generally lags behind. The digital economy within western urban agglomerations has low development due to the following reasons: the economic base limits the ability to construct a complete digital economic infrastructure in support of its development, and the rapid iteration and updates of the industry necessitates a significant number of skilled professionals to provide support. However, the city's restricted level of urban livability and economic growth restrict its attractiveness to high-end individuals.

### Comprehensive evaluation of green technology innovation system

In this paper, the entropy weight method is used to calculate the comprehensive score of green technology innovation of each city, and the results are weighted and averaged to obtain the comprehensive score ranking of green technology innovation of urban agglomerations in China, and the grades are divided according to the proportion (see Table 6).

**Table 6:** Comprehensive score and classification of green technology innovation system in urban agglomerations of China.

Urban agglomeration	Year				Total score	Classification of grades
	2011	2014	2017	2020		
Pearl River delta	0.091	0.079	0.163	0.148	0.120	High
The Beijing-Tianjin-Hebei	0.103	0.102	0.111	0.106	0.106	High
The Yangtze river delta	0.095	0.084	0.121	0.114	0.104	High
Shandong Peninsula	0.026	0.026	0.036	0.037	0.031	Higher up
Chengyu	0.023	0.024	0.042	0.030	0.030	Higher up
Guangdong, Fujian and Zhejiang coastal	0.020	0.017	0.033	0.031	0.025	Medium
Central Yunnan	0.020	0.020	0.028	0.029	0.024	Medium
Guanzhong Plain	0.023	0.018	0.025	0.021	0.022	Medium
Yangtze River Delta	0.017	0.015	0.025	0.023	0.020	Medium
Central plains	0.013	0.012	0.023	0.020	0.017	Lower
Harbin-changjiang	0.015	0.016	0.015	0.017	0.016	Lower
South Central Liaoning	0.021	0.014	0.015	0.012	0.016	Lower
Central Guizhou	0.008	0.013	0.016	0.017	0.013	Lower
The north slope of Tianshan Mountain	0.012	0.010	0.013	0.009	0.011	Lower
Beibu Gulf	0.006	0.010	0.015	0.010	0.010	Low
Jinzhong	0.007	0.007	0.008	0.010	0.008	Low
Lanxi	0.006	0.006	0.008	0.009	0.007	Low
Hubao Eyu	0.004	0.006	0.008	0.010	0.007	Low
Ningxia along the yellow	0.003	0.003	0.006	0.007	0.005	Low

There was an upward trend from 2011 to 2017, followed by a slight decrease from 2017 to 2020.

Overall, the level of green technology innovation in urban agglomerations varied greatly between 2011 and 2020. The degree of development in green technology innovation in individual urban agglomerations from 2011 to 2020 differed, each with distinct characteristics. The disparity between the Pearl River Delta urban agglomeration with the highest overall score and the Ningxia along the Yellow River Urban Agglomeration with the lowest score is 0.115, indicating a significant gap. The Pearl River Delta City cluster, the Beijing-Tianjin-Hebei City cluster, the Yangtze River Delta City cluster, and the Shandong Peninsula City cluster occupied the top four positions among the 19 major city clusters in green technology innovation ratings. The Pearl River Delta, Beijing-Tianjin-Hebei, and Yangtze River Delta are the three most influential urban agglomerations in China. These findings are consistent with Qing et al.^54^. Their development outpaces other agglomerations, and they boast more advanced innovations in green technology. The high score of green technology innovation in the Shandong Peninsula urban agglomeration can be attributed to its favourable geographical location and industrial structure, creating a conducive environment for innovation. This advantage is absent in many other urban agglomerations, resulting in lower innovation scores.

### Global statistical analysis of coupling coordination degree

After analysing Table 7, the findings indicate that there are noticeable spatial differentiation characteristics regarding the degree of coupling coordination in the TG system in Chinese urban agglomerations.

**Table 7:** Descriptive statistics of coupled coordination degree of TG systems in urban agglomerations in China.

Urban agglomeration	Year				Total score	Coupling coordination classification	Coupling coordination relation
	2011	2014	2017	2020			
Pearl River delta	0.337	0.399	0.498	0.459	0.423	Borderline disorder	Basic synchronization
Yangtze river delta	0.304	0.346	0.404	0.384	0.359	Mild disorder	GTI lags slightly
The Beijing-Tianjin-Hebei	0.247	0.307	0.343	0.335	0.308	Mild disorder	GTI lags slightly
Shandong Peninsula	0.180	0.219	0.249	0.246	0.223	Moderate dissonance	GTI generally lags behind
Guangdong, Fujian and Zhejiang coastal	0.175	0.202	0.240	0.233	0.213	Moderate dissonance	GTI generally lags behind
Ningxia along the yellow	0.170	0.200	0.233	0.220	0.206	Moderate dissonance	GTI generally lags behind
Central Yunnan	0.149	0.194	0.219	0.208	0.192	Severe disorder	GTI is lagging behind
Tianshan north slope	0.191	0.184	0.197	0.187	0.190	Severe disorder	GTI is lagging behind
Chengyu	0.142	0.185	0.228	0.201	0.189	Severe disorder	GTI is lagging behind
The Yangtze River Delta	0.125	0.161	0.196	0.184	0.166	Severe disorder	GTI is lagging behind
Central plains	0.121	0.155	0.191	0.198	0.166	Severe disorder	GTI is lagging behind
Liaoning Central and Southern	0.148	0.164	0.172	0.156	0.160	Severe disorder	GTI is lagging behind
Hubao Eyu	0.108	0.154	0.172	0.188	0.156	Severe disorder	GTI is lagging behind
Beibu Gulf	0.108	0.155	0.182	0.153	0.150	Severe disorder	GTI is lagging behind
Harbin-changjiang	0.127	0.152	0.161	0.158	0.150	Severe disorder	GTI is lagging behind
Guanzhong Plain	0.126	0.149	0.167	0.149	0.148	Severe disorder	GTI is lagging behind
Central Guizhou	0.091	0.158	0.159	0.169	0.144	Severe disorder	GTI is lagging behind
Jinzhong	0.110	0.143	0.150	0.151	0.138	Severe disorder	GTI is lagging behind
Lanxi	0.096	0.132	0.151	0.156	0.134	Severe disorder	GTI is lagging behind

The majority of urban agglomerations in China show a severe disorder in the coupling coordination degree of TG systems, with only a few displaying a moderate or mild disorder. The coupling coordination degree of electrical power transmission and generation systems in all urban agglomerations ranged from 0.134 to 0.423. Thirteen urban agglomerations were found to be in a severely dysfunctional state. Formatting adhered to common academic conventions, including consistent citation and quoting. The Pearl River Delta urban agglomeration had the highest coupling coordination level, followed by the Yangtze River Delta urban agglomeration and the Beijing-Tianjin-Hebei urban agglomeration, both in a mild disordered state. These findings are consistent with Zhang and Yin^8^, Zhang et al.^30^, the digital economy is underdeveloped and lacks the capacity to efficiently integrate its digital components into the green technology innovation system. There is still scope for enhancing the coordinated advancement of the digital economy and innovative green technology. Various factors contribute to the disharmony in the coupling coordination degree of TG system in urban agglomerations. The discrepancy in the coupling coordination degree of the TG system in certain urban agglomerations is due to insufficiencies in the city's conditions, which fail to provide adequate space for the digital economy, consequently inhibiting its development. Proper planning is needed to promote the development of a harmonious integration between the digital economy and green technology innovation.

The number of urban agglomerations in a state of green technology innovation development that lags behind, as presented by the TG system, is higher than those in a state of basic synchronicity as per the TG system. The TG system indicates that the urban agglomerations, which are behind the curve in digital economy development, are predominantly situated in the western and northern regions of China. The coupling coordination of the TG system in China's urban agglomerations indicates a spatial distribution pattern with high degrees in the east and south and low degrees in the west and north. The coastal city cluster in the east has shown advanced development in the digital economy, implementing it in urban development and construction. The region has also adopted effective measures to support talent acquisition, green development, and livelihood guarantee. The coupling coordination degree of the TG system is relatively high in certain western urban agglomerations, particularly on the Northern Slope of the Tianshan Mountain. The city cluster situated on the north slope of the Tianshan Mountain is a significant location for population and resource gatherings in northwest China, and a crucial city cluster in the development of China's Silk Road Economic Belt. In recent years, the urban agglomeration of Tianshan Mountain's Northern Slope has invested heavily in digital economy, particularly in the construction of digital economy carrier projects. However, issues persist in the application of digital technology. Generally, the coupling and coordination of the TG system in the western urban agglomeration of China is poorer than in the eastern urban agglomeration. However, these findings are consistent with Han et al.^55^ being an erstwhile industrial hub, the urban conglomeration situated in the northeast of the country has insufficient space for application and realisation of value, resulting in the digital economy development lagging behind that of green technology innovation, with a lower level of coupling coordination than the southern urban agglomerations.

### Global trend surface analysis of coupling coordination degree

**Figure 2:**
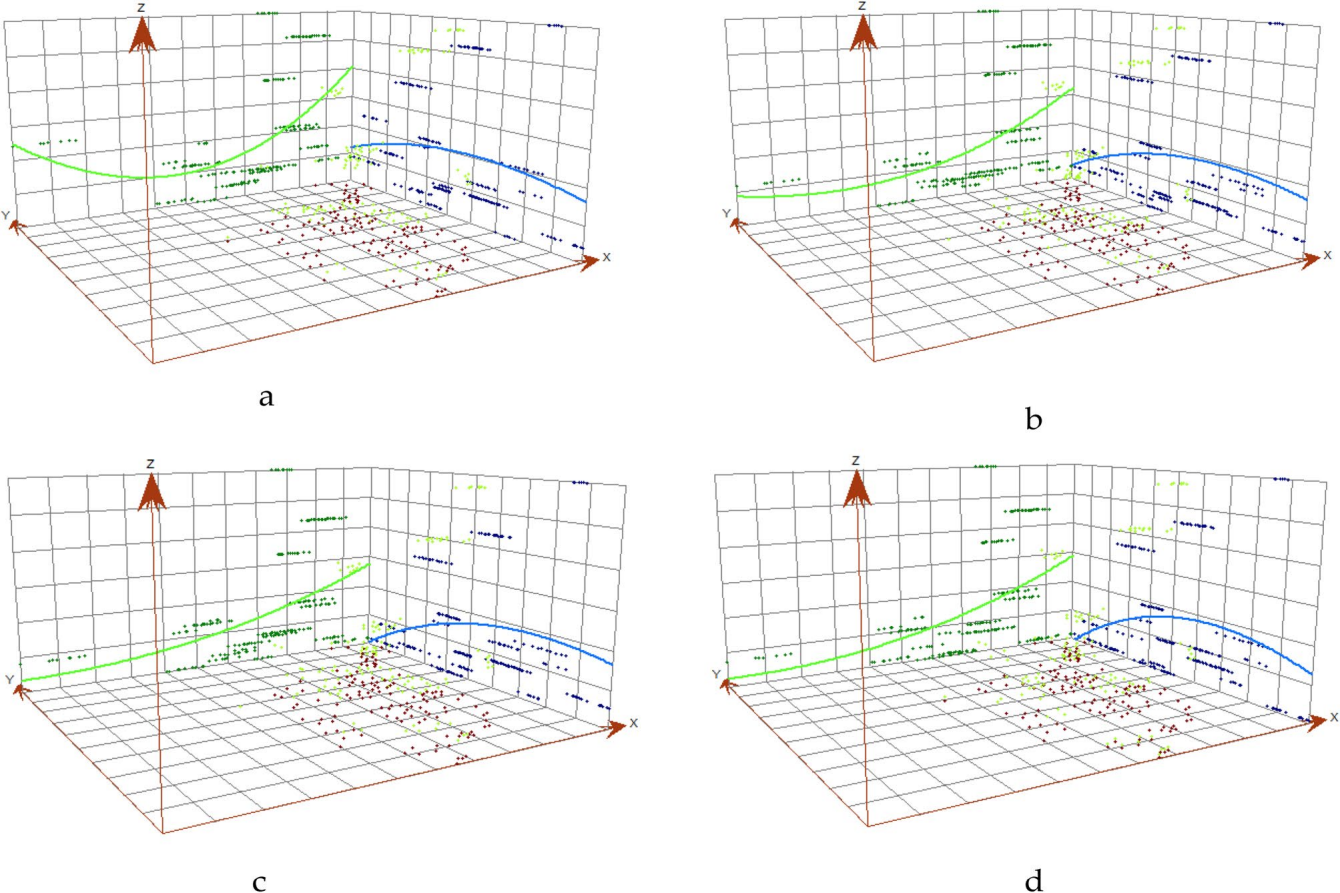
Spatial trends of TG system coupling coordination degree in 2011, 2014, 2017 and 2020. (**a**) In 2011. (**b**) In 2014. (**c**) In 2017. (**d**) In 2020.

The spatial trend analysis charts depicting the coupled and coordinated development scores of TG systems in urban agglomerations in 2011, 2014, 2017 and 2020 were drawn using ArcGIS10.2. Please refer to Fig. 2 for further details. The diagram shows the trend analysis for the spatially coupled and coordinated development score of the TG system in urban agglomeration. The East is indicated on the X-axis and the North on the Y-axis. The green and blue lines respectively illustrate the East–West and North–South levels of the combined and coordinated development score of the TG system in urban agglomeration.

Overall, the coupling coordination degree of the TG system in the urban agglomeration exhibits a spatial trend of "low in the west and high in the east" in the east–west direction. This suggests that the coupling coordination degree of the TG system in the eastern urban agglomeration is generally better than that in the western urban agglomeration. In the north–south direction, there is a gradual increase in the coupling coordination degree of the TG system in the urban agglomeration, with high levels in the south and low levels in the north. This smooth transition indicates a spatial trend of increasing coordination from north to south.

From a projection arc perspective, the coupling coordination degree projection arc of the TG system is noticeably steeper in the eastern urban agglomeration than in the western region. This suggests that the difference in coupling coordination degree of the TE system is greater in the eastern urban agglomeration than in the western region. The north–south projection of the spatial trend chart that demonstrates the coordination of the TG system within urban agglomerations is relatively smooth and indicates a minimal spatial difference in the coupled coordination degree of the TG system during the north–south transition.

Upon closer evaluation of the time series, the differences in the coupling and coordination degrees of the TG system in urban agglomerations are generally uniform. Over the entire time period considered, the level of coupling coordination in the TG systems of urban agglomerations in the eastern and southern regions of China (including the Beijing-Tianjin-Hebei urban agglomeration and the Yangtze River Delta urban agglomeration) is generally higher than that found in the western and northern regions of China (such as the Jinzhong urban agglomeration and the Lanci urban agglomeration).

### Spatio-temporal evolution analysis of coupling coordination degree

**Figure 3:**
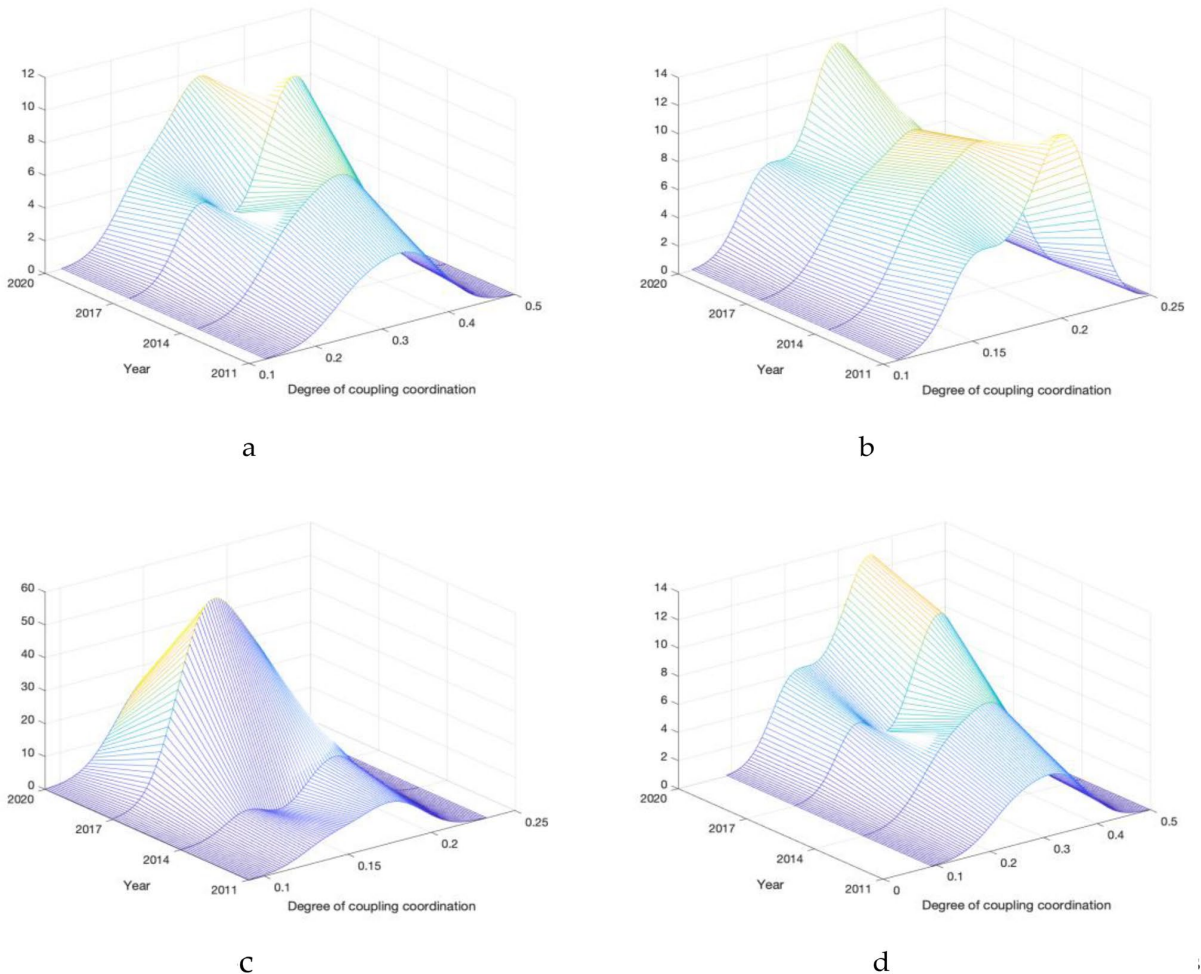
Development level distribution of coupling coordination degree of urban agglomerations in China. (**a**) Optimizing and Upgrading Urban ag-glomerations. (**b**) Developing and expanding urban agglomerations. (**c**) Fostering and developing urban agglomerations. (**d**) Nineteen urban agglomerations.

Figure 3 illustrates the progress of TG system coupling and coordination degree in 19 urban agglomerations in China throughout the study period. Technical term abbreviations will be explained when first used. Bias will be avoided, and the language will be clear, formal, and objective. Consistent formatting features and citation style will be maintained throughout the text, which will be grammatically correct and free of spelling and punctuation errors. Analyzing the distribution, there was a slight rightward trend in the center of the kernel density function of the 19th urban agglomeration, indicating a slight improvement in the TG system's coupling coordination degree during the study period. Secondly, the distribution curve of the urban agglomeration is wide, implying a significant gap in the internal coupling coordination of the TG system. Looking at the distribution pattern, the wave crest of the urban agglomeration is higher, and this indicates a healthy and sustainable developmental trend of the urban agglomeration with high coupling coordination of the TG system. From a standpoint of distribution flexibility, there is a trailing characteristic that suggests a convergence trend in the coupling coordination degree of the TG system as a whole, with differences gradually decreasing. Secondly, the main lateral peaks in urban agglomerations are relatively far apart, indicating a polarization phenomenon in the coupling coordination degree of the TG system within these agglomerations. Overall, the coupling coordination degree of the TG system displays traits of "horizontal fluctuation changes, absolute difference enlargement, and noticeable polarization phenomena". As the absolute difference continues to increase and polarization becomes more pronounced in the TG system coupling coordination degree, the focus should be on narrowing the gap and promoting regional coordinated development in the subsequent development policy formulation.

Figure 3 and Table 7 display the Kernel density estimation outcomes alongside the dynamic evolution features of the coupling coordination level among the 19th urban agglomerations and three others with diverse development policies. These results depict the overall distribution pattern of the urban coupling coordination degree development level and allow for grasping the dynamic characteristics through comparative analysis across different periods. Technical abbreviations such as "Kernel density estimation" are explained upon initial use. The language is clear and objective, employing a formal register devoid of jargon, filler words, and ambiguous terms. The text adheres to conventional structure and format guidelines, follows consistent citation, footnote, and referencing protocols. The grammar, spelling, and punctuation are error-free. The position of the distribution, shape of the main peak, ductility, and number of peaks in the density curve have been analysed.

Firstly, let us examine the distribution. Throughout the study period, the nuclear density distribution curves of the three urban agglomeration types in China shifted to the right, and the final curve is positioned towards the far right. In 2020, there is a certain gap between the curve's central point and 2011, signalling that the country's development level and the three urban agglomerations have improved steadily.

Secondly, let us consider the distribution pattern. The peak height of the national nuclear density distribution curve has increased, suggesting a decrease in the dispersion level of development of coupling coordination degree in urban agglomerations nationwide, while the development level of coupling coordination degree in most urban agglomerations has increased. Due to significant disparities in the development foundations and policy support of various regional urban agglomerations, there is an evident polarization in the degree of coupling coordination between these urban agglomerations. The trend of nuclear density distribution in the optimized and enhanced urban agglomeration is consistent with that of the entire country. Further, the height of the main peak is increasing, suggesting that the absolute difference in coordination development between the optimized and enhanced urban agglomeration and the entirety of the country is decreasing.

Thirdly, during the study period, a bimodal phenomenon was evident across all areas of the country, including the three major urban agglomerations, with a noticeable disparity between the main and lateral peak drop. There was also a polarization phenomenon in the development of the coupling coordination degree of urban agglomerations within the region, with some urban agglomerations exhibiting clear advantages in development level. The development and growth of urban agglomerations in China have shifted from a unimodal to a bimodal pattern, and the phenomenon of polarization is becoming increasingly apparent. The urban cluster for farming and growth has remained unimodal, and there is no apparent trend of polarization.

Overall, an analysis of the urban agglomeration coupling coordination degree shows characteristics of "steady improvement of level and obvious polarization phenomenon" both at the national level and regional levels. To address the polarization in the coupling coordination degree among urban agglomerations, development policies should prioritize narrowing the gap and promoting regional coordinated development (See Table 8).

**Table 8:** Dynamic evolution characteristics of coupling coordination degree of urban agglomerations in China.

Region	Location of distribution	Main peak distribution pattern	Number of wave peaks
Optimize and upgrade urban agglomerations	Right shift	The height goes down and the width goes up	Single or double peak
Developing and expanding urban agglomerations	Right shift	The height goes down and the width goes up	Single or double peak
Fostering and developing urban agglomerations	Right shift	The height goes down and the width goes up	Single or double peak
Nineteen urban agglomerations	Right shift	The height goes down and the width goes up	Single or double peak

### Analysis of spatial correlation characteristics of coupling coordination degree

#### Multi-distance spatial clustering analysis

**Figure 4:**
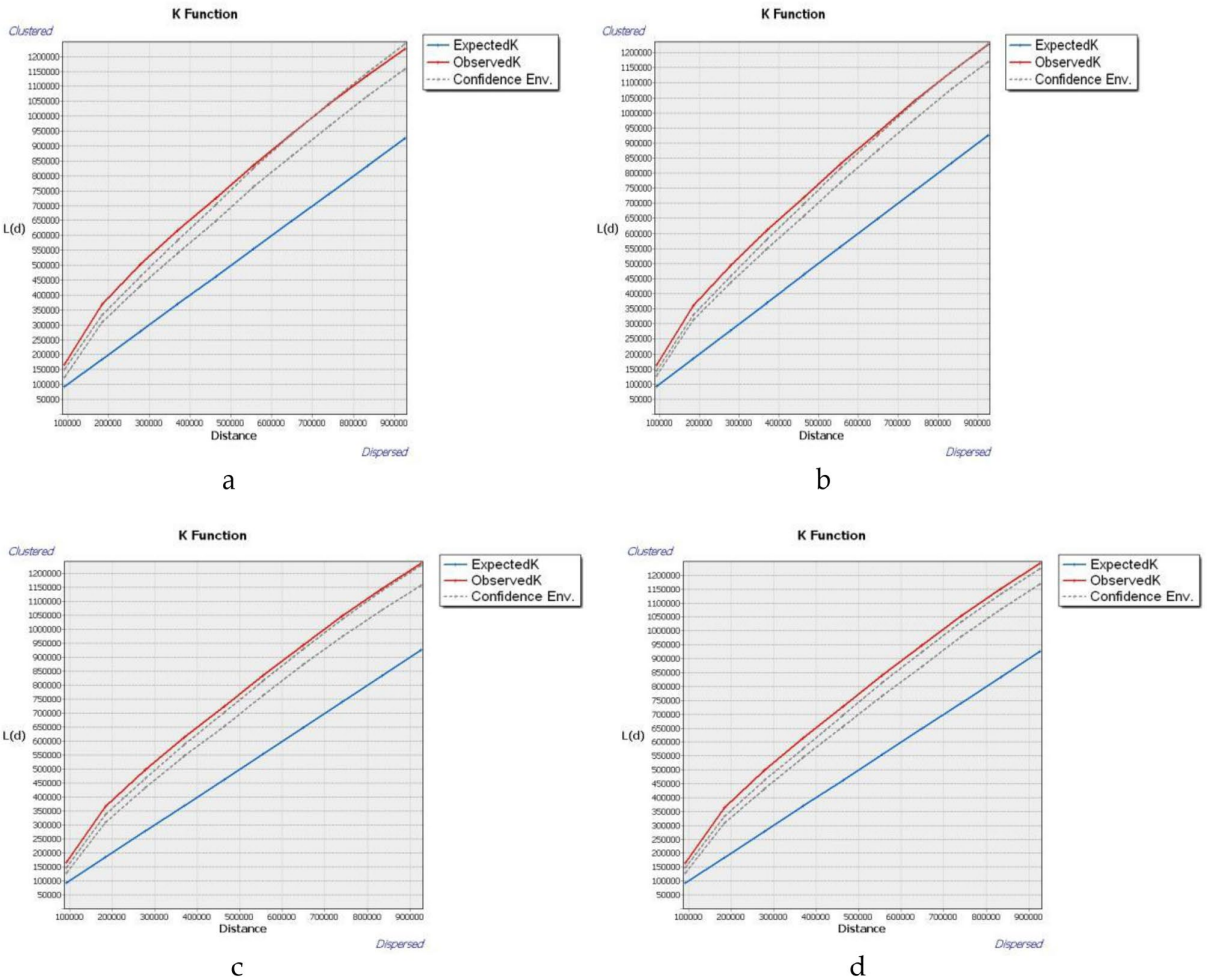
Ripley's K function curve of coupling coordination degree of TG system. (**a**) In 2011. (**b**) In 2014. (**c**) In 2017. (**d**) In 2020.

Based on the analysis of the Ripley's K function for the TG system within the urban agglomeration of China (Fig. 4), it is evident that all the L(d) function curves, except for the ones at the end distance scales, fall outside the confidence interval range between 2011 and 2017. The findings suggest that the distribution of the TG system in Chinese urban agglomerations between 2011 and 2017 increasingly approached the agglomeration distribution typical of the model distribution. Nevertheless, as of 2020, all L(d) function curves for the TG system of urban agglomerations in China exceeded the confidence interval, indicating that the spatial pattern of the TG system in Chinese urban agglomerations now conforms to the agglomeration distribution. On the whole, the spatial correlation characteristics of thermal generation (TG) systems in Chinese urban agglomerations underwent a gradual shift from model-dependent distribution to agglomeration distribution between 2011 and 2020.

#### Spatial autocorrelation analysis

The distribution of coupling coordination degree for TG systems differs significantly across various urban agglomerations. Consequently, the presence of spatial correlation can be established by evaluating the global Moran's I, the outcomes of which are displayed in Table 6. The study demonstrates that all global Moran's I values for the years 2011, 2014, 2017, and 2020 exceed 0 and successfully pass the 1% significance test. This confirms the presence of a significant positive spatial correlation amongst urban agglomerations (See Table 9).

**Table 9:** Moran's I calculation results of TG system coupling coordination degree in urban agglomeration of China.

Variables of interest	In 2011	In 2014	In 2017	In 2020
Moran's I	0.961	0.950	0.934	0.940
Z score	35.709	35.294	34.759	34.949
*P* value	0.000	0.000	0.000	0.000

### Analysis on the factors influencing the spatio-temporal evolution of the coupling coordination degree of digital economy and green technology innovation in urban agglomerations in China

According to the relevant measurements, the coordination degree of the TG system within China's urban agglomerations presents spatial and temporal variations. In order to investigate the impact of the TG system's subsystem coupling coordination on the TG system of Chinese urban agglomerations, this study incorporates the actual development of China's digital economy and green technology innovation. Relevant indicators are selected, and a driving factor model is constructed to determine the degree of TG system coupling coordination^56^.

#### Factor detector analysis

The paper employs SPSS 26.0 software to optimally discretize each factor, followed by utilizing the factor detector in the geographical detector to scrutinize and evaluate the impact of each factor on the coupling coordination degree of TG system within China's urban agglomerations. The outcomes exhibit that the identified factors in 2011, 2014, 2017, and 2020 have all passed the 1% significance test (See Table 10).

**Table 10:** Coupled driving factors of TG system in urban agglomerations in China and the detection results of geographical detectors.

Driving force	Driving factors	Index	Unit	2011	2014	2017	2020
Economic sustainability	Economic development	Regional GDP per capita	Yuan	0.235	0.378	0.430	0.411
	Rationalization of the industrial structure	The ratio of output value of tertiary industry to secondary industry	–	0.217	0.227	0.438	0.379
Policy support	Regulation by government	The proportion of expenditure in the general budget of local finance to GDP	–	0.428	0.086	0.110	0.202
	Development of Finance	Outstanding loans as a share of GDP	–	0.391	0.361	0.274	0.342
Digital driving force	Degree of digitization	Number of broadband Internet access users per 100 people	People	0.212	0.275	0.159	0.151
	Digital Talent	Proportion of computer software and software industry employees	–	0.200	0.186	0.187	0.284
Technical support	Advances in technology	Research expenditure	Ten thousand yuan	0.212	0.278	0.254	0.264
	Green invention	Number of green inventions applied annually	Number	0.646	0.645	0.675	0.655
Talent creativity	Senior talent	Expenditure on education	Ten thousand yuan	0.239	0.246	0.240	0.239
	Human capital	Logarithm of the resident population at the end of the year	–	0.380	0.387	0.442	0.472

The technical support force exerts the greatest influence on the degree of coupling coordination within the TG system. This supportive force remained steady, fluctuating minimally, between 2011 and 2020. Within the realm of 10 factors, the driving force behind green innovation holds the most significant influence, displaying fluctuations ranging between 0.646 and 0.675. This empirical outcome is consistent with the earlier study by Cioffi et al.^57^, China is bolstering the dominant position of enterprises, elevating their role in technological innovation, and invigorating various innovation entities. Implement and refine a set of compulsory technical standards with the aim of stimulating innovation in green technologies within enterprises. The Chinese Government has continued to introduce policies and initiatives to support the technological innovation of high-tech enterprises in order to enhance their dominant position. It has also strengthened the protection of intellectual property rights and supported digital financial services to create a favourable environment for green technology innovation.

The economic durability significantly impacts the coupling coordination degree of the TG system. Between 2011 and 2017, the index for economic development rose from 0.235 to 0.430, and the index for industrial structure rationalization increased from 0.217 to 0.438, with a subsequent downward trend from 2017 to 2020. The digital economy is a vital and essential mode of economic growth in the pursuit of high-quality development. China is actively promoting digital industrialization and the integration of digital technologies with the real economy to foster high-quality economic development.

The talent creativity moderately impacts the coordination of the TG (technological gap) system, with the creativity of the human capital level gradually increasing from 0.380 to 0.472 between 2011 and 2020. This empirical outcome is consistent with the earlier study by Huang et al.^9,10^, human capital has a positive impact on the coupled coordination of the TG system, China has consistently enhanced its talent acquisition, training, and remuneration system, advanced the implementation of its talent acquisition programme, and reinforced the development of crucial personnel, proficient in scientific, technological, and innovative fields, primarily focused on digital economies. Conversely, the proficiency of highly skilled workers indicates minimal and consistent variations. China has consistently enhanced its talent acquisition, training, and remuneration system, advanced the implementation of its talent acquisition programme, and reinforced the development of crucial personnel, proficient in scientific, technological, and innovative fields, primarily focused on digital economies.

The impact of policy support on the coupling coordination degree of TG system is minimal. Within the period of 2011–2020, government regulation exhibited pronounced fluctuations, attaining a peak of 0.428 and a nadir of 0.086. This pattern correlates significantly with China's policy orientation during this period, while the level of financial development demonstrates relatively gradual fluctuations. China will maximise the effectiveness of policy regulation and enhance policy support for digital economy in order to promote green technology innovation in line with regional development features. It will establish a favourable external environment for investment promotion and ensure institutional protection and development opportunities for technological innovation, digital information technology, economic progress, and ecological environment improvement.

The impact of the digital driving force on the coordination degree of TG system is the least. From 2014 to 2020, the level of digitalization has been declining, while the level of digital talent has exhibited considerable fluctuation. China aims to augment the construction of digital infrastructure, research and develop digital technologies, elevate the state of digital development, foster digital-enabled creativity, and foster deeper levels of physical digitalization. Develop an information model platform for the urban agglomeration and establish a standardised digital map of public infrastructure within urban areas. Ensure the timely and accurate capture of real-time data from physical fields and integrate it into a digital resource library for urban infrastructure^58^.

#### Interactive detector analysis

The geographic detector's interactive detector analyzes pairwise interactions among ten indicators that affect the spatial differentiation of TG system coupling coordination in Chinese urban agglomerations. This analysis excludes subjective evaluations and adheres to a clear, concise, and objective writing style, as well as proper grammar and spelling conventions. Technical term abbreviations are explained as needed, and the text is organized in a logical manner with causal connections between ideas. Additionally, the text uses precise vocabulary and regular citation and footnote formatting. The pairwise relationship between impact factors of TG system coupling coordination in China's urban agglomerations is enhanced, with two-factor and nonlinear enhancement, indicating no independent or weakened relationship. The results from the table indicate that the spatial differentiation pattern of the 19th urban agglomerations in China is a result of the interaction and coordination of multiple factors.

In 2011, the region with the highest interactive influence had a per capita GDP and education expenditure as high as 0.760. Additionally, the interaction value of the ratio of expenditure in the general budget of local finance to GDP and the ratio of loan balance to GDP with education expenditure was 0.728. It is apparent that there is a significant correlation between expenses in education and other contributing factors, potentially due to China's active development in economy, prioritization of education investment, and increased emphasis on talent training, resulting in the accelerated coupling and coordinated development of the TG system.

In 2014, the highest value of interactive influence was defined as the ratio between the annual number of green inventions applied for and the expenditure within the local budget to GDP, with a value as high as 0.833. This was followed by the logarithm of the regional per capita GDP and the year-end permanent population, with an interaction value of 0.751. Technical terms are clearly explained when introduced. The text is written in a clear and objective manner, complying with conventional structure and formal register. There are no grammatical or spelling errors. The interactive impact value of the number of environmentally friendly inventions implemented during the medieval era and other relevant factors is generally high. This may be attributed to the Chinese government's and state's significant focus on innovation in green technology, the implementation of innovative system changes, the enactment of legislation specifically geared towards quickening the process of green patent examination, the creation of an information governance system for free green patents, and the establishment of a platform for sharing green patents. The promotion of the coupled and coordinated development of the TG system is advocated for.

In 2017, the application rate of environmentally-friendly inventions was highest in the year with the most significant interactive impact. The logarithm of the population at the end of that year accounted for 0.782 of the application rate, with the regional per capita GDP and logarithm of the population following close behind, with an interaction value of 0.755. Among the ten impact factors, the number of green inventions applied during the year and the logarithm of the permanent population at the end of the year had the greatest interactive influence on other factors. This relationship may be attributed to China's demographic dividend and the acceleration of urbanisation, accompanied by the growth of human capital. As the supporting role of green technology innovation becomes increasingly significant, it is crucial to note the importance of environmentally friendly approaches in driving progress.

In 2020, the region with the highest interactive influence had a proportion of per capita GDP and loan balance in GDP as high as 0.749. Following closely behind were the number of green inventions applied annually and education expenditure, with an interactive value of 0.742. Technical abbreviations will be explained when used for the first time. The text adheres to conventional academic structure, clear objective language, formal register, and precise word choice. Spelling and grammar follow British English norms, and the writing is free from grammatical errors, spelling mistakes and punctuation errors. Among the ten influencing factors, the greatest interactions with other factors are observed in the regional per capita GDP, the number of annual applications of green inventions, and the logarithm of the permanent resident population at the end of the year. These factors could be related to China's efforts towards developing a market-oriented system for green technology innovation to stimulate high-quality economic growth.

## Conclusions and implications

### Discussion

The coupling model of digital economy and green technology innovation constructed in this paper calculates the development and coupling degree of digital economy and green technology innovation in China from 2011 to 2020, and promotes the coordinated development of digital economy and green technology innovation based on the coupling model. The calculation results are basically consistent with the literature^6,30,40,50^. The calculation results show that the overall degree of coupled and coordinated development of digital economy and green technology innovation in China maintains a continuous improvement, but the spatial difference is significant. With the rapid development of the digital economy, the overall improvement of green technology innovation is also crucial. It is also necessary to play the spatial linkage effect to optimize the coordinated regional development; to create a multi-level development pattern to lead high-quality development. Realizing the coupled synergistic development of digital economy and green technology innovation at a higher level should be realized as soon as possible. These conclusions provide a theoretical basis for countries to formulate policies to promote the coordinated development of the digital economy and green technological innovation, with a view to building a digital ecological civilization rich in green wisdom.

### Conclusion

Based on data from 200 prefecture-level cities within China's 19 urban agglomerations spanning 2011–2020, this study employs a TOPSIS entropy weight method, coupling coordination degree model, multi-distance spatial cluster analysis, and Moran's I to investigate the coupling coordination degree of the TG system, along with its spatio-temporal evolution and spatial correlation characteristics. Technical term abbreviations are explained when first used, and a clear, concise writing style is utilized throughout to ensure maximum comprehensibility. And using the Geographic Detector model to investigate its influencing factors, the following conclusions have been drawn:

This paper presents a theoretical framework for the TG system, which explores the interactions between the digital economy and green technology innovations. The study analyses coupling coordination, coupling coordination relationships, and the degree of such interactions. From 2011 to 2020, there has been significant progress in the development of the digital economy and green technology innovations in urban agglomerations, resulting in varying degrees of improvement from the baseline year of 2011. 68% of urban agglomerations experienced severe disorders. Among them, the urban agglomerations of Shandong Peninsula and the coastal areas of Guangdong, Fujian, and Zhejiang exhibit a relatively high degree of coupling and coordination between their transportation and resource systems, thanks to their favourable geographic positions. Conversely, urban agglomerations in western inland regions, such as Lanxi and Jinzhong, experience limited coupling and coordination of transportation and resource systems, due to transportation geography and other factors. As an old industrial base, the city clusters located in the Northeast region lag behind in the development of digital economy compared to green technology innovation due to the lack of sufficient space for application and value realization, and their coupling coordination is lower than that of the city clusters in the South.

This paper identifies three types of urban agglomerations in China, guided by diverse policy backgrounds. These types include the optimization and upgrading of urban agglomerations, development and expansion of urban agglomerations, and cultivation of urban agglomerations. Technical terms will be explained when first used, and the paper adheres to conventional academic structure with clear, concise and necessary information in simple sentences. The language is formal, objective and value-neutral while avoiding biased or figurative language, colloquial words, and unnecessary jargon. Passive tone and impersonal construction will be used, and first-person perspectives will be avoided unless necessary. The paper follows consistent citation and footnote style, adheres to grammatical correctness and uses precise word choices that convey the meaning precisely. The digital economy and green technology innovation scores of economically developed urban agglomerations, such as urban clusters, are primarily enhanced and optimised. Furthermore, the Kernel density estimation outcomes and dynamic trends in the levels of coupling and coordination among the three varieties of urban agglomerations with diverse development policies differ, with the overall trend indicating “steady improvement and evident polarization.”

This study conclusively demonstrates the significance of exploring the coupling and coordinated development of digital economy and green technology innovation as a crucial avenue for future research. The first rank among them is held by the correlation degree of technical support and coupling coordination degree. This could be attributed to China's persistent efforts to enhance the dominant position of enterprises, bolster their role in technological innovation, and enthuse innovation subjects' vitality. We will revise, enhance, and enforce a set of obligatory technical standards to stimulate businesses to innovate in green technology. The reinforcement of intellectual property protection is linked to promoting digital finance and additional services, thereby creating a favourable environment for innovating in green technologies.

### Deficiencies and future research

Although this study has important theoretical and practical significance, there are still some deficiencies to be addressed in future research. (1) This paper only selected the data of 2011, 2014, 2017 and 2020 as the nodes for dynamic research, which is not comprehensive enough; in future research, a more scientific approach should be adopted to study each time node. (2) This paper only reveals the influence mechanism of each factor on the coordinated development of digital economy and green economy from the perspective of 5 driving forces; in future research, the influence mechanism of other factors on the coordinated development of digital economy and green economy should be explored. (3) The indicators for measuring digital economy and green technology innovation are incomplete; in future research, we will consider adding relevant indicators to comprehensively measure digital economy and green technology innovation.

## Data Availability

It can be obtained directly from the corresponding author.
